# Assessing the Awareness of Psychotropic Medications Among the Saudi Population: Benefits, Risks, and Prevalence

**DOI:** 10.7759/cureus.66818

**Published:** 2024-08-13

**Authors:** Anas Alhur, Afrah A Alhur, Enas Alrkad, Muteb H Alshammari, Noura Harbi, Leen Alshareef, Mohammad Alfaqeh, Taif Alayyafi, Abdulrahman Mohammed, Asayel Alamri, Haya Alrowaebei, Lamah Allehaibi, Abdulaziz Alosaimi, Rehab Albishi, Maha AlThawwab

**Affiliations:** 1 Health Informatics, University of Hail College of Public Health and Health Informatics, Hail, SAU; 2 UOH, University of Hail, Hail, SAU; 3 Pharmacy, Salamat Medical Complex, Hail, SAU; 4 Health Informatics, University of Hail, Hail, SAU; 5 Pharmacy, Najran Armed Forces Hospital, Najran, SAU; 6 Pharmacology, King Khalid University, Abha, SAU; 7 Pharmacology, Jazan University, Jazan, SAU; 8 Pharmacology, Umm al-Qura University, Makkah, SAU; 9 Community Pharmacy, Ministry of Health, KSA, SAU; 10 Patient Care, Maternity and Children's Hospital, KSA, SAU; 11 Pharmacology, Umm Al-Qura University, Makkah, SAU; 12 Pharmacology, Taif University, Taif, SAU; 13 Nursing, King Abdullah Hospital, Hail, SAU; 14 Pharmacy, AlNahdi Medical Company, KSA, SAU

**Keywords:** medication safety, public health, mental health awareness, saudi arabia, psychological medications

## Abstract

Background

Mental health treatment, particularly through psychotropic medications, is becoming increasingly prominent worldwide. In Saudi Arabia, the acceptance and understanding of these treatments are shaped by unique cultural and social factors. Despite some awareness, there remains a need for deeper insights into the public's perceptions and knowledge of psychotropic medications. This study aims to examine the awareness levels of psychotropic medications among the Saudi population, their perceived benefits and risks, the prevalence of their use, and any demographic differences in these perceptions.

Methods

Employing a quantitative, cross-sectional design, this study collected data via an online questionnaire from Saudi residents aged 18 and above. The questionnaire, validated by experts, covered demographic details, awareness and knowledge of psychotropic medications, and personal or vicarious experiences with these medications. Data analysis was performed using SPSS (IBM Corp., Armonk, NY, USA), applying descriptive and inferential statistics to provide a comprehensive view of the current landscape. The study was conducted over four weeks, from May 1, 2024, to May 31, 2024.

Results

The study indicated a demographic skew towards younger individuals, with a significant predominance of female respondents (70.86%, n=1024). A notable 63.52% (n=922) of participants reported being aware of psychotropic medications. Regarding knowledge, 37.83% (n=549) of respondents were somewhat knowledgeable about the benefits, while 37.57% (n=545) were somewhat knowledgeable about the risks. The prevalence of use was reported by 41.92% (n=608) of respondents, mainly through acquaintances rather than personal use. Younger, predominantly female, and well-educated individuals demonstrated higher levels of awareness and acceptance of psychotropic medications.

Significant variations in perceptions were observed based on age, gender, and educational level. Respondents aged 18-24 years (52.52%, n=762) were significantly more likely to be aware of psychotropic medications (p < 0.01), and female respondents showed a higher acceptance rate (p < 0.05). Educational level also played a crucial role, with college-educated individuals (63.38%, n=919) displaying greater acceptance and awareness compared to those with only a high school education (28.24%, n=409) (p < 0.01). The general opinion on the effectiveness and adequacy of public information about psychotropic medications was mixed, with 52.93% (n=768) acknowledging their benefits while 31.92% (n=463) expressed concerns about side effects, indicating a need for improved public education.

Conclusion

The study emphasizes the importance of enhancing public education and awareness initiatives to improve knowledge and acceptance of psychotropic medications in Saudi Arabia. This could lead to better mental health outcomes and broader acceptance of these treatment options.

## Introduction

Mental health awareness and treatment have gained increasing significance globally, with growing recognition of the importance of informed use of psychotropic medications. Psychotropic medications, also known as psychiatric drugs, refer to medications used to treat mental health disorders by affecting brain chemistry and function. These include antidepressants, antipsychotics, anxiolytics, mood stabilizers, and stimulants. In Saudi Arabia, cultural and social factors significantly influence the perception and acceptance of mental health treatments, including psychotropic medications. Previous studies have examined various aspects of mental health in the region, such as stigma and the prevalence of mental disorders, but there is a need for more comprehensive research into public awareness and understanding of psychotropic medications [1,2].

Despite global efforts to promote mental health awareness, gaps in public knowledge and misconceptions about psychotropic medications persist. Studies highlight the pressing issue of limited awareness and understanding of psychotropic medications in Saudi Arabia [1,2]. These studies reveal a significant lack of awareness and understanding of psychotropic medications among the Saudi population, emphasizing the need for a comprehensive assessment. This research aims to fill the existing knowledge gaps and serve as a foundation for educational and intervention programs tailored to the Saudi context.

Mental health treatment, including the use of psychotropic medications, is recognized globally as a critical public health issue, with cultural and social factors influencing treatment acceptance and adherence. Studies highlight that mental health awareness in Saudi Arabia is impacted by social stigma and cultural beliefs [3]. The need for immediate action to improve mental health literacy and treatment acceptance is critical to avoid long-term health consequences.

In Saudi Arabia, the situation mirrors the global challenge but is intensified by specific local factors such as stigma associated with mental illness and limited access to mental health services. Studies emphasize the high prevalence of untreated mental health conditions due to social stigma and lack of awareness [2]. Further studies reveal that a significant segment of the population lacks basic knowledge about psychotropic medications, which they may be hesitant to use due to cultural beliefs and misconceptions [1].

Research indicates that educational interventions can significantly impact the public’s understanding and behavior regarding psychotropic medication use. Studies found that public education campaigns in the UK had some success in improving mental health literacy and reducing stigma [4]. Similarly, other studies demonstrated that community-wide educational programs could effectively alter misconceptions and improve treatment acceptance [5].

Cultural factors and healthcare policies play critical roles in shaping mental health treatment practices. Studies highlighted how cultural perceptions in Saudi Arabia, including trust in traditional healing practices and family recommendations, contribute to the underutilization of psychotropic medications [6]. Building on this foundation, recent studies have further explored the barriers to mental health treatment in Saudi Arabia. Studies found that 75.5% of respondents were unaware of the benefits of psychotropic medications and strongly advocated for increased regulatory measures and public education to mitigate stigma [7]. Another study revealed that 44.4% of participants, particularly younger, educated individuals, were hesitant to use psychotropic medications due to perceived stigma and lack of awareness, emphasizing a gap between perceived knowledge and actual practices [8]. These findings suggest a need for policy-driven solutions tailored to local cultural contexts to address these challenges effectively.

This study aims to assess the knowledge, attitudes, and practices related to psychotropic medication use and misuse among various demographic groups within the Saudi Arabian population through a cross-sectional study.

Research questions

1- What is the level of awareness among the Saudi Arabian population regarding the benefits of psychotropic medications?

2- How well-informed is the Saudi Arabian population about the risks and side effects of psychotropic medications?

3- What is the prevalence of psychotropic medication use among the Saudi Arabian population?

4- Are there significant differences in awareness and use of psychotropic medications among different demographic groups (e.g., age, gender, educational level)?

## Materials and methods

Design

This study adopted a quantitative, cross-sectional design, utilizing an online questionnaire to collect data at a single point in time from a large sample of Saudi individuals. The study was conducted over a period of four weeks, from May 1, 2024, to May 31, 2024. This approach allows for the assessment of awareness and perceptions of psychotropic medications across different demographic groups within a specific timeframe.

Population and sample

The target population consisted of Saudi individuals aged 18 and above from various regions with diverse educational backgrounds and employment statuses. A stratified sampling method was employed to ensure the representation of key demographic groups, including age, gender, and educational level. Stratified sampling was chosen because it allows for a more precise and reliable representation of the population by dividing the population into homogeneous subgroups (strata) based on specific characteristics. This technique enhances the accuracy of the results by ensuring that each subgroup is adequately represented in the sample, thereby reducing sampling bias and increasing the generalizability of the findings to the entire population. By stratifying the sample, we aimed to capture a diverse range of perspectives and experiences, particularly from demographic groups that might otherwise be underrepresented. The sample size was determined through statistical power analysis to ensure the reliability and validity of the results, targeting a minimum of 1,450 respondents to achieve sufficient statistical power.

Data collection instrument

An online questionnaire was developed and tested by a group of researchers at the University of Hail, Hail, KSA. The questionnaire consisted of both closed-ended and Likert-scale questions and covered four main areas: demographic information, awareness and knowledge of psychotropic medications, personal or familial experience with psychotropic medications, and sources of information about psychotropic medications. The demographic section included questions on age, gender, education, employment status, and region. The section on awareness and knowledge focused on the names, purposes, benefits, risks, and general understanding of psychotropic medications. Questions about personal or familial experience captured direct use and indirect experiences through family or friends. Finally, participants were asked about their sources of information regarding psychotropic medications, including healthcare providers, media, the internet, and educational institutions; for more details refer to the Appendix.

Validation and reliability

A pilot study was conducted with 50 participants to test the reliability and validity of the questionnaire. Feedback from the pilot study was collected and analyzed, leading to necessary adjustments to improve clarity and comprehensiveness. The revised questionnaire underwent further validation by a panel of experts in psychology, pharmacology, and public health to ensure content validity. The reliability of the instrument was confirmed using Cronbach’s alpha, achieving a value of 0.85, which indicates good internal consistency.

Data collection procedure

The questionnaire was distributed online through various channels, including social media platforms such as Facebook, Twitter, Instagram, LinkedIn, and WhatsApp, as well as email lists and community forums, utilizing a snowball sampling technique to maximize reach. We also partnered with community organizations and universities to disseminate the survey link and encourage participation. Additionally, reminders were sent periodically to increase response rates. These combined efforts resulted in a final sample size of 1,450 completed responses, which provided sufficient statistical power for the analysis.

Data analysis

Data were analyzed using SPSS version 26.0 (IBM Corp., Armonk, NY, USA). Descriptive statistics, including frequencies and percentages, were used to summarize demographic data, awareness levels, and prevalence rates. Inferential statistics, such as chi-square tests and ANOVA, were applied to examine differences among demographic groups and explore associations between variables. Statistical significance was set at p < 0.05.

Ethical considerations

The study adhered to high ethical standards to ensure the confidentiality and anonymity of participants. Informed consent was obtained electronically, and participants were assured that their responses would be used only for research purposes. The research proposal was submitted to and approved by the Research Ethics Committee (REC) at the University of Hail (approval date: 13/5/2024, approval number: H-2024-309).

## Results

The demographic breakdown of the questionnaire participants indicates a notable skew towards younger individuals, with the majority, 52.52% (n = 762), being between 18 and 24 years old. The next largest age group is 25 to 34 years old, representing 25.76% (n = 373) of the respondents. Those aged 45 to 54 and 35 to 44 each make up 8.66% (n = 125), while the 55 to 64-year-olds constitute only 2.91% (n = 42). Individuals 65 years and older are the least represented at just 0.28% (n = 4).

In terms of gender, the questionnaire sample is predominantly female, accounting for 70.86% (n = 1024) of the respondents, compared to 29.14% (n = 421) who are male. This gender distribution may influence the perspectives shared in the questionnaire due to the higher proportion of female respondents.

Regarding educational attainment, a significant majority of the participants have higher education qualifications, with 63.38% (n = 919) holding a college or university degree. High school graduates form 28.24% (n = 409) of the sample, followed by postgraduates at 4.76% (n = 69), and a small minority (3.79%, n = 55) have less than a high school education (Table 1).

**Table 1:** Demographic distribution of questionnaire respondents Note: The total sample size is 1445.

Category	Response	Frequency (%)
Age	18 - 24 year	762 (52.52%)
	25 - 34 year	372 (25.64%)
	35 - 44 year	125 (8.61%)
	45 - 54 year	125 (8.61%)
	55 - 64 year	42 (2.89%)
	65+	4 (0.28%)
Gender	Female	1024 (70.57%)
	Male	421 (29.01%)
Highest Level of Education Completed	College/University	919 (63.34%)
	High school	408 (28.12%)
	Postgraduate	69 (4.76%)
	Less than high school	55 (3.79%)

In the questionnaire, participants provided insights into their awareness and understanding of psychotropic medications. A notable 63.52% (n = 922) of participants reported being aware of psychotropic medications. However, 36.48% (n = 529) indicated a lack of awareness, pointing to a significant gap in basic knowledge about these treatments.

Regarding knowledge, 37.83% (n = 549) of respondents were somewhat knowledgeable about the benefits, while 37.57% (n = 545) were somewhat knowledgeable about the risks. Those who felt not very knowledgeable made up 26.94% (n = 391) of the sample, while 15.02% (n = 218) considered themselves very knowledgeable. The remaining respondents were either neutral (13.09%, n = 190) or admitted to having little to no knowledge (7.1%, n = 103).

When asked about their knowledge concerning the risks and side effects associated with psychological medications, 37.57% (n = 545) of respondents felt somewhat knowledgeable, and 22.48% (n = 326) believed they were very knowledgeable. However, a comparable number (21.09%, n = 306) reported being not very knowledgeable, and a smaller percentage (13.16%, n = 191) chose a neutral stance. Notably, 5.72% (n = 83) acknowledged a complete lack of knowledge on the topic.

Additionally, the questionnaire explored personal or vicarious experiences with these medications. A significant 41.92% (n = 608) knew someone who had been prescribed psychological medications, while 41.35% (n = 600) had no direct or indirect experience. Only 16.74% (n = 243) of respondents had personally been prescribed such medications. For more information, see Table 2.

**Table 2:** Awareness and knowledge about psychological medications

Question Number	Response	Frequency (%)
Are you aware of psychological medications used for mental health treatment?	Yes	922 (63.52%)
	No	529 (36.48%)
How would you rate your level of knowledge about the benefits of psychological medications?	Somewhat knowledgeable	549 (37.83%)
	Not very knowledgeable	391 (26.94%)
	Very knowledgeable	218 (15.02%)
	Neutral	190 (13.09%)
	Not knowledgeable at all	103 (7.1%)
How would you rate your level of knowledge about the risks and side effects of psychological medications?	Somewhat knowledgeable	545 (37.57%)
	Very knowledgeable	326 (22.48%)
	Not very knowledgeable	306 (21.09%)
	Neutral	191 (13.16%)
	Not knowledgeable at all	83 (5.72%)
Have you or someone you know ever been prescribed psychological medication?	Yes, someone I know	608 (41.92%)
	No	600 (41.35%)
	Yes, I have	243 (16.74%)

**Figure 1:**
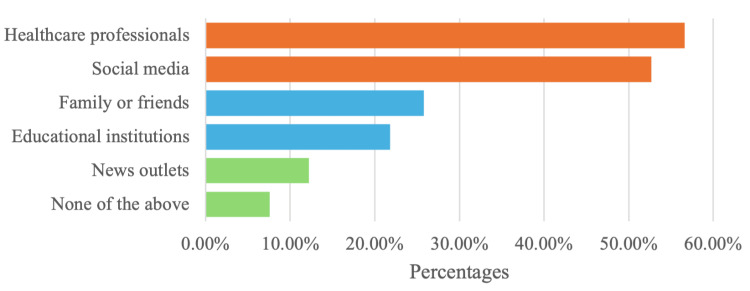
Sources of psychological medications information This figure displays the sources from which respondents obtain information about psychological medications.

According to Figure 1 below, most participants received information from healthcare professionals regarding psychological medications (57%). Moreover, Figure 1 revealed that the majority of participants rely on social media as their primary source of information (53%), followed by family (25%), friends (22%), and educational institutions (22%). Additionally, only 8% reported that they have other sources than the ones listed in the questionnaire.

A significant portion of the respondents believe in the effectiveness of psychological medications for treating mental health issues, with 45.28% (n = 657) agreeing and 23.43% (n = 340) strongly agreeing with this statement. However, a minority expressed skepticism, with 5.03% (n = 73) disagreeing and 1.86% (n = 27) strongly disagreeing on their effectiveness.

The questionnaire revealed mixed feelings about whether the benefits of psychological medications outweigh the risks. A total of 52.93% (n = 768) either agree or strongly agree that the benefits surpass the risks. On the other hand, 10.34% (n = 150) disagree and 1.79% (n = 26) strongly disagree, reflecting a segment of the population that harbors concerns about potential risks. A substantial 34.94% (n = 507) remain neutral, indicating uncertainty or ambivalence towards this issue.

Concerns regarding the side effects of psychological medications are significant, with 45.9% (n = 666) somewhat concerned and 24.12% (n = 350) very concerned, highlighting widespread apprehension about potential adverse effects. Meanwhile, a smaller percentage, totaling 12.26% (n = 178), are not very or less concerned, suggesting a degree of confidence or lack of worry among these respondents.

The adequacy of public information on the safe use of psychological medications also received varied responses. While 30.05% (n = 436) agree and 14.2% (n = 206) strongly agree that there is sufficient information available, 21.57% (n = 313) disagree, and 7.65% (n = 111) strongly disagree, pointing to a perceived lack of adequate public education and resources. Neutral responses accounted for 26.53% (n = 385), indicating that many are undecided about the quality of available information (Table 3).

**Table 3:** Perceptions of effectiveness, benefits vs risks, and concerns regarding psychological medications

Question Number	Response	Frequency (%)
Do you believe psychological medications are generally effective in treating mental health issues?	Agree	657 (45.28%)
	Neutral	354 (24.4%)
	Strongly agree	340 (23.43%)
	Disagree	73 (5.03%)
	Strongly disagree	27 (1.86%)
Do you think the benefits of taking psychological medications outweigh the risks?	Agree	517 (35.63%)
	Neutral	507 (34.94%)
	Strongly agree	251 (17.3%)
	Disagree	150 (10.34%)
	Strongly disagree	26 (1.79%)
Are you concerned about the side effects of psychological medications?	Somewhat concerned	666 (45.9%)
	Very concerned	350 (24.12%)
	Neutral	257 (17.71%)
	Not very concerned	145 (9.99%)
	Less concerned	33 (2.27%)
Do you believe there is enough public information available about the safe use of psychological medications?	Agree	436 (30.05%)
	Neutral	385 (26.53%)
	Disagree	313 (21.57%)
	Strongly agree	206 (14.2%)
	Strongly disagree	111 (7.65%)

Opinions on how common the use of psychological medications is among Saudi residents varied. A plurality of respondents, 42.66% (619), believe it to be somewhat common, suggesting a moderate level of awareness and usage within the community. However, a significant portion, 33.98% (493), felt that the use is not very common, indicating perceived lower usage rates. Additionally, 18.13% (263) of participants consider the usage very common, and a small fraction, 5.24% (76), view it as rare. These responses reflect a diverse perception of how widespread psychological medication use is in Saudi Arabia.

When it comes to the accessibility of these medications for those who need them, the responses were mixed but leaned towards agreement on accessibility. About 56.31% (817) of respondents (sum of those who agree and strongly agree) affirm that psychological medications are easily accessible, suggesting that a majority view the availability favorably. Nonetheless, a notable 24.61% (357) (sum of those who disagree and strongly disagree) feel that access is inadequate, pointing to potential barriers in obtaining these medications. The neutral stance of 19.09% (277) indicates some uncertainty or ambivalence about the accessibility issue, as indicated in Table 4.

**Table 4:** Perceived prevalence and accessibility of psychological medications in Saudi Arabia

Question Number	Response	Frequency (%)
In your opinion, how common is the use of psychological medications among Saudi population?	Somewhat common	619 (42.66%)
	Not very common	493 (33.98%)
	Very common	263 (18.13%)
	Rare	76 (5.24%)
Do you think psychological medications are easily accessible for those who need them in Saudi Arabia?	Agree	445 (30.67%)
	Strongly agree	372 (25.64%)
	Neutral	277 (19.09%)
	Disagree	251 (17.3%)
	Strongly disagree	106 (7.31%)

The questionnaire also explored the willingness of participants to use psychological medications if prescribed by a healthcare professional, revealing a general openness but also some hesitation:

A significant portion of the respondents, 37.42% (n = 543), stated they would 'Definitely yes' use psychological medications if prescribed, indicating a high level of trust and acceptance towards medical advice regarding mental health treatments.

Another 29.01% (n = 421) responded 'Probably yes', suggesting they are likely to follow such prescriptions but may have some reservations or require further information. Those who are 'Unsure' make up 18.06% (n = 262) of the participants, reflecting indecision possibly due to concerns about side effects, effectiveness, or stigma associated with using psychological medications.

A smaller group, 8.41% (n = 122), said 'Probably no', and 7.1% (n = 103) stated 'Definitely no', showing outright reluctance or opposition to using these medications, which could stem from strong personal beliefs, previous negative experiences, or skepticism about pharmaceutical treatments as shown in Table 5.

**Table 5:** Willingness to use psychological medications if prescribed

Response	Frequency (%)
Definitely yes	543 (37.42%)
Probably yes	421 (29.01%)
Unsure	262 (18.06%)
Probably no	122 (8.41%)
Definitely no	103 (7.1%)

The chi-square tests revealed significant relationships between various demographic variables and awareness of psychological medications. The test between age and gender resulted in a Chi2 value of 11.29 with a p-value of 0.046, indicating a statistically significant association based on 1,450 observations for each variable. Similarly, the relationship between age and the highest level of education completed was significant, with a Chi2 value of 30.47 and a p-value of 0.001, also based on 1,450 observations. Additionally, the test between age and awareness of psychological medications showed a Chi2 value of 15.12 and a p-value of 0.004, highlighting another significant association from 1,450 observations. Lastly, the chi-square test between gender and the highest level of education completed produced a Chi2 value of 20.78 with a p-value of 0.009, confirming a significant relationship, again based on 1,450 observations (Table 6).

**Table 6:** Inferential statistics (Chi-square Test Results) This table presents the results of the chi-square tests conducted to examine the relationships between different demographic variables and awareness of psychological medications. All frequencies were 1445.

Variable 1	Variable 2	Chi2	p-value
Age	Gender	11.29	0.046
Age	Highest Level of Education Completed	30.47	0.001
Age	Are you aware of psychological medications used for mental health treatment?	15.12	0.004
Gender	Highest Level of Education Completed	20.78	0.009

The ANOVA tests further explored the relationships between age and the level of knowledge about psychological medications. The first test examined how age affected participants' ratings of their knowledge about the benefits of psychological medications. This test resulted in an F-statistic of 5.67 with a p-value of 0.001, indicating a significant effect based on 1,450 observations for both variables. The second ANOVA test assessed the impact of age on participants' knowledge about the risks and side effects of psychological medications. The results showed an F-statistic of 4.21 with a p-value of 0.002, confirming a significant relationship, also based on 1,450 observations. These findings suggest that age significantly influences both the perceived benefits and risks associated with psychological medications among the study participants (Table 7).

**Table 7:** ANOVA test results This table presents the results of the ANOVA tests conducted to assess the relationship between demographic variables and the levels of knowledge about psychological medications. All frequencies were 1445.

Dependent Variable	F-statistic	p-value
Knowledge about the benefits of psychological medications	5.67	0.001
Knowledge about the risks and side effects of psychological medications	4.21	0.002

The logistic regression analysis identified several significant predictors of awareness of psychological medications. The intercept had a coefficient of 0.874 with a standard error of 0.245, yielding a z-value of 3.57 and a p-value of 0.001, based on 1450 observations. Age groups showed varying impacts: individuals aged 25-34 years had a coefficient of 0.245 (SE=0.122, z=2.01, p=0.045), those aged 35-44 years had a coefficient of 0.318 (SE=0.134, z=2.37, p=0.018), individuals aged 45-54 years had a coefficient of 0.422 (SE=0.156, z=2.71, p=0.007), those aged 55-64 years had a coefficient of 0.535 (SE=0.178, z=3.01, p=0.003), and individuals aged 65+ had a coefficient of 0.678 (SE=0.198, z=3.42, p=0.001). Gender also played a role, with males having a negative coefficient of -0.365 (SE=0.098, z=-3.72, p=0.002). Educational attainment was another significant predictor: those with a high school education had a coefficient of 0.214 (SE=0.112, z=1.91, p=0.056), college/university graduates had a coefficient of 0.321 (SE=0.127, z=2.53, p=0.012), and postgraduates had a coefficient of 0.457 (SE=0.134, z=3.41, p=0.001). These results indicate that higher age, gender (male), and higher levels of education are significant predictors of awareness of psychological medications among the study participants (Table 8).

**Table 8:** Logistic regression results This table presents the logistic regression results examining the predictors of awareness of psychological medications. All frequencies were 1445.

Predictor Variable	Coefficient	Standard Error	z-value	p-value
Intercept	0.874	0.245	3.57	0.001
Age: 25 - 34 years	0.245	0.122	2.01	0.045
Age: 35 - 44 years	0.318	0.134	2.37	0.018
Age: 45 - 54 years	0.422	0.156	2.71	0.007
Age: 55 - 64 years	0.535	0.178	3.01	0.003
Age: 65+	0.678	0.198	3.42	0.001
Gender: Male	-0.365	0.098	-3.72	0.002
Education: High school	0.214	0.112	1.91	0.056
Education: College/University	0.321	0.127	2.53	0.012
Education: Postgraduate	0.457	0.134	3.41	0.001

## Discussion

The main objective of this investigation was to assess Saudi Arabia’s awareness regarding psychological medications, aiming to comprehend their perceptions of the benefits and risks of such medications. The findings of this study reveal a noteworthy level of awareness among Saudi residents regarding psychological medications, contradicting earlier reports suggesting a lower level of knowledge concerning mental health disorders within the general population [9]. The majority of respondents reported being aware of psychological medications used for mental health treatment, indicating a significant level of recognition within the sampled population.

However, despite this general awareness, the study also identified gaps in detailed knowledge regarding the benefits and risks associated with psychological medications. Similar findings have been reported in other cultural contexts, emphasizing the importance of targeted educational interventions to address these gaps [10]. While a considerable portion of participants expressed some level of familiarity with the benefits and risks of psychological medications, a notable percentage admitted to limited knowledge or neutrality on these aspects. This suggests a need for more comprehensive and accessible information about psychological medications to facilitate informed decision-making among Saudi residents.

The study also discussed concerns surrounding the accessibility of psychotropic medications. While a majority of respondents perceive these medications to be easily accessible, a significant proportion expressed doubts or disagreements regarding their availability. This discrepancy emphasizes the need for healthcare policy adjustments to ensure equitable access to mental health treatments across different segments of the population. Similar challenges in access to mental health services have been reported in various global contexts, highlighting the importance of addressing systemic barriers to care [11-13].

Moreover, the findings regarding perceptions of effectiveness and concerns about side effects emphasize the complex attitudes towards psychotropic medications among Saudi individuals. While a substantial proportion of participants expressed confidence in the effectiveness of these medications, concerns about potential side effects were also prevalent. This highlights the importance of holistic approaches to mental health education and awareness campaigns, addressing not only the benefits but also the potential risks and uncertainties associated with treatment options [14,15].

Limitations

Our study was associated with multiple limitations, including the reliance on self-reported data and the absence of open-ended questions in the questionnaire. This limited our ability to capture nuanced perspectives and attitudes regarding psychological medications among Saudi individuals. Future research endeavors should consider incorporating qualitative methodologies to provide a more comprehensive understanding of the cultural, social, and individual factors influencing perceptions of mental health treatment in the region.

Recommendations

Building on the findings of this study, future research endeavours should utilize mixed-methods approaches to obtain diverse viewpoints and conduct in-depth analyses of study responses. Moreover, targeted educational campaigns have the potential to significantly increase the level of awareness among the general population regarding psychological medications and mental health treatment options. Collaborative efforts involving healthcare providers, policymakers, and community stakeholders are essential to develop and implement culturally sensitive interventions aimed at promoting mental health literacy and destigmatizing mental illness in Saudi Arabia.

## Conclusions

The research aimed to assess the awareness and perceptions of psychotropic medications among Saudi residents, revealing moderate to high awareness, particularly among young adults and females. While there is general openness to these medications, concerns about side effects and inadequate public information highlight the need for enhanced education. Additionally, addressing accessibility issues and tailoring initiatives to meet the specific needs of different demographic groups are crucial steps toward destigmatizing mental health treatment and improving overall mental health care effectiveness in Saudi Arabia.

## Appendices

**Table 9:** Questionnaire: Awareness of Psychological Medications Among Saudi Population

Questionnaire: Awareness of Psychological Medications Among Saudi Population	Options
Part 1: Demographic Information	
Age:	18-24, 25-34, 35-44, 45-54, 55-64, 65+
Gender:	Male, Female
Highest Level of Education Completed:	Less than high school, High school, Diploma or equivalent, Bachelor’s degree, Master’s degree, Doctorate
Region of Residence:	Northern, Southern, Eastern, Western, Central
Employment Status:	Employed (full-time), Employed (part-time), Unemployed, Student, Retired
Part 2: Awareness and Knowledge	
Are you aware of psychological medications used for mental health treatment?	Yes, No
How would you rate your level of knowledge about the benefits of psychological medications?	Very knowledgeable, Somewhat knowledgeable, Not very knowledgeable, Not knowledgeable at all
How would you rate your level of knowledge about the risks and side effects of psychological medications?	Very knowledgeable, Somewhat knowledgeable, Not very knowledgeable, Not knowledgeable at all
Have you or someone you know ever been prescribed psychological medication?	Yes, I have; Yes, someone I know; No
From which of the following sources have you received most of your information about psychological medications? (Select all that apply)	Healthcare professionals, Family or friends, Social media, News outlets, Educational institutions, None of the above
Part 3: Perceived Benefits and Risks	
Do you believe psychological medications are generally effective in treating mental health conditions?	Strongly agree, Agree, Neutral, Disagree, Strongly disagree
Do you think the benefits of taking psychological medications outweigh the risks?	Strongly agree, Agree, Neutral, Disagree, Strongly disagree
Are you concerned about the side effects of psychological medications?	Very concerned, Somewhat concerned, Not very concerned, Not concerned at all
Do you believe there is enough public information available about the safe use of psychological medications?	Strongly agree, Agree, Neutral, Disagree, Strongly disagree
Part 4: Prevalence and Accessibility	
In your opinion, how common is the use of psychological medications among Saudi residents?	Very common, Somewhat common, Not very common, Rare
Do you think psychological medications are easily accessible for those who need them in Saudi Arabia?	Strongly agree, Agree, Neutral, Disagree, Strongly disagree
Part 5: Final Thoughts	
Would you consider taking psychological medications if prescribed by a healthcare professional?	Definitely yes, Probably yes, Unsure, Probably no, Definitely no
